# Healthy working life expectancy at age 50 for people with and without osteoarthritis in local and national English populations

**DOI:** 10.1038/s41598-022-06490-3

**Published:** 2022-02-14

**Authors:** Marty Lynch, Milica Bucknall, Carol Jagger, Ross Wilkie

**Affiliations:** 1 School of Medicine, Keele University, Newcastle under Lyme, ST5 5BG UK; 2 MRC Versus Arthritis Centre for Musculoskeletal Health and Work, University of Southampton, Southampton, SO17 1BJ UK; 3 Population Health Sciences Institute, Newcastle University, Newcastle upon Tyne, NE4 5PL UK

**Keywords:** Epidemiology, Public health

## Abstract

Retirement ages are rising in many countries to offset the challenges of population ageing, but osteoarthritis is an age-associated disease that is becoming more prevalent and may limit capacity to work until older ages. We aimed to assess the impact of osteoarthritis on healthy working life expectancy (HWLE) by comparing HWLE for people with and without osteoarthritis from ages 50 and 65 nationally and in a local area in England. Mortality-linked data for adults aged ≥ 50 years were used from six waves (2002–13) of the English Longitudinal Study of Ageing and from three time points of the North Staffordshire Osteoarthritis Project. HWLE was defined as the average number of years expected to be spent healthy (no limiting long-standing illness) and in paid work (employment or self-employment), and was estimated for people with and without osteoarthritis and by sex and occupation type using interpolated Markov chain multi-state modelling. HWLE from age 50 years was a third lower for people with osteoarthritis compared to people without osteoarthritis both nationally (5.68 95% CI [5.29, 6.07] years compared to 10.00 [9.74, 10.26]) and in North Staffordshire (4.31 [3.68, 4.94] years compared to 6.90 [6.57, 7.24]). HWLE from age 65 years for self-employed people with osteoarthritis exceeded HWLE for people without osteoarthritis in manual or non-manual occupations. Osteoarthritis was associated with a significantly shorter HWLE. People with osteoarthritis are likely to have significantly impaired working ability and capacity to work until older ages, especially in regions with poorer health and work outcomes.

## Introduction

As life expectancy increases and populations age in many countries, people are expected to remain in paid work until they are older^1^. Poor health and lack of appropriate job opportunities are major reasons for early retirement, work absence and reduced productivity; it is unclear if people in later working-age life (age ≥ 50) are able to work for longer^2–4^. Population ageing is associated with increasing prevalence of age-associated diseases including musculoskeletal disorders^5^. In the UK in 2018, musculoskeletal problems accounted for 27.8 million days of sickness absence, which was 19.7% of all 141.4 million working days lost to sickness absence^6^. Osteoarthritis is the most common musculoskeletal joint condition in adults, the fastest increasing health condition globally and a leading cause of disability^7,8^. Prevalence and general practice consultation incidence of osteoarthritis sharply increase in age groups over 50^9–11^. A quarter of the UK population aged 50–65 have consulted their general practice for osteoarthritis treatment^11,12^. Incidence and prevalence of osteoarthritis varies across UK regions^9^. Indirect costs of osteoarthritis such as through work absence and reduced productivity at work far exceed direct costs (which are driven by healthcare costs, particularly joint replacement)^13,14^. UK economy production losses due to indirect costs of osteoarthritis are estimated to exceed £3.2 billion per year^13,14^. The high prevalence of osteoarthritis in adults aged 50 and over and increasing prevalence with age indicates a need to understand the relationship between osteoarthritis and health and work at population level and the potential for this large group of people to extend their working life under national and local conditions^13,15,16^.

Healthy working life expectancy (HWLE) at age 50 is the average number of years a person is expected to be healthy and in work from age 50^17^. HWLE overall in England has been previously calculated as 9.42 years from age 50 with regional differences that are likely to be linked to varying health burdens, employment quality, deprivation levels, and economic competitiveness^3,18^. Across Europe, available estimates suggest HWLE is highest (and similar to England) in countries facing fewer challenges to “active ageing” in employment, living, environmental, and societal participation contexts (e.g. Finland, Denmark, Germany, Sweden, Czech Republic, Israel, and Switzerland)^17,19,20^. However, due to methodological differences, numerical international comparisons cannot be easily made between recently published HWLE estimates for groups of European countries and earlier estimates published with the introduction of the indicator^17,19^. The Sullivan method (in which health expectancies are calculated from simple formulae using lifetables) is useful for comparing different time points as it is not subject to attrition, but the approach relies on production of life tables and is therefore restricted in the subgroups it can compare.

Building on our previous study of HWLE in England^3^, this study aimed to estimate HWLE for people with and without osteoarthritis, overall and by sex and occupation type. It is one of the first to examine HWLE together with specific chronic disease. The study also investigated the potential to examine variation in HWLE among those with and without osteoarthritis by geographical region. This was done using local data as insufficient sample size prevented estimation of regional HWLE associated with osteoarthritis status using national data. HWLE estimates are presented from age 50 as well as at age 65 to isolate expected healthy working years beyond what was—until 2018—the long-standing UK State Pension age for men. Estimates of HWLE at age 65 indicate the average duration of healthy working life during the years of age that are becoming newly ineligible for the State Pension, and suggest whether work is likely to meet the financial gap left by increasing State Pension age. As an example of a local area in England, HWLE was examined in North Staffordshire.

## Methods

### Data sources

Data from a nationally representative longitudinal survey (English Longitudinal Survey of Ageing; ELSA) were used to calculate HWLE in those with and without osteoarthritis at national and small regional level. Data from the North Staffordshire Osteoarthritis Project (NorStOP) were used to examine HWLE at a local level.

#### ELSA

Data from ELSA waves 1–6 (2002/3–2012/13) were used^21^. ELSA has been described in detail elsewhere^22^. Briefly, ELSA collects longitudinal survey data from a representative sample of community-dwelling adults aged 50 and over in England. ELSA introduced refreshment samples of additional participants at waves 3, 4, and 6 to maintain a representative sample of adults aged 50 and over despite deaths, cohort ageing, and attrition. Linked mortality data from the National Health Service Central Register are available up to ELSA wave 6 (2012/13).

#### NorStOP

NorStOP data are representative of England’s North Staffordshire region with linked medical records data^23^. Full details of NorStOP are available elsewhere^23,24^. NorStOP collected longitudinal survey and medical record data from adults aged 50 years and over registered at eight General Practices in North Staffordshire. Three cohorts were invited to participate in NorStOP and complete baseline questionnaires (NorStOP 1: March 2002–September 2002; NorStOP 2: July 2002–July 2003; and NorStOP 3: February 2004–May 2005). Baseline respondents were sent follow-up questionnaires at three years and six years after baseline. Data from all three NorStOP cohorts were combined for this analysis. Medical records for consenting participants were obtained from 12 months prior to the start of the cohort’s baseline recruitment period up to six-year follow-up, or up to three-year follow-up if there was no response to follow-up questionnaires. Forty per cent of the population of North Staffordshire live in rural areas while 99% of Stoke-on-Trent (the largest city in North Staffordshire) is urban. Thirty per cent of Stoke-on-Trent neighbourhoods are in the most deprived decile in England measured by relative deprivation in income, housing, education, employment, health, crime, and living environment. However, 10 neighbourhoods, mostly in North Staffordshire, are in the most affluent decile. Relative to England, the resident population has less ethnic diversity; 91% identify as White, with Asian/Asian British the next most common ethnic group comprising 9% of the population of Stoke-on-Trent.

#### Identification of health, work, and osteoarthritis status

In ELSA, health and work statuses were self-reported in each survey wave. Health was defined by the presence or absence of limiting long-standing illness^2,3^, obtained from a combination of two survey items: “Do you have any long-standing illness, disability or infirmity?” and, if so, “(Does this/Do these) illness(es) or disability(ies) limit your activities in any way?”. Work was defined as participation in paid work or self-employment within the month preceding the interview.

In ELSA, osteoarthritis status was a time-dependent variable defined as self-report of ever having received a doctor diagnosis for osteoarthritis (or arthritis of an unknown type) identified from survey responses. Individuals were considered to have osteoarthritis if, at that wave or at an earlier wave, they self-reported having been told by a doctor that they have osteoarthritis. (As a gradually progressive chronic condition, osteoarthritis will always remain present following self-reported doctor diagnosis^25^.) Arthritis of an unknown type was assumed to be osteoarthritis as this is the most common form of arthritis^26^. Self-reported no-osteoarthritis statuses were carried backwards into earlier missing values.

In NorStOP, health (that is, no long-standing limiting illness) was defined as the absence of limitations due to physical health (one question) or emotional problems (one question). The physical health question was “During the past 4 weeks, have you had any of the following problems with your work or other regular daily activities as a result of your physical health?” with sub-items: “Were limited in the kind of work or other activities”, and “Accomplished less than you would like”. The mental health question was “During the past 4 weeks, have you had any of the following problems with your work or other regular daily activities as a result of any emotional problems (such as feeling depressed or anxious)?” with sub-items: “Didn’t do work or other activities as carefully as usual”, and “Accomplished less than you would like”. Individuals were considered to have poor health if they responded ‘yes’ to at least one of the physical or mental health sub-items and were healthy otherwise. Work was defined from self-reported employment status in the questionnaires: ‘employed’ at baseline and three-year follow-up; and ‘in full-time paid work’, ‘in part-time paid work but not retired’ or ‘in part-time paid work and partly retired’ at six-year follow-up. Osteoarthritis status in NorStOP was a time-independent variable defined as having osteoarthritis during or within 12 months before the study period (NorStOP 1: 2002–2008; NorStOP 2: 2002/3–2008/9; NorStOP 3: 2004/5–2010/11) and identified through medical record Read codes. General practitioners in the study used the hierarchical Read code system to code the reasons for clinical encounters in primary care consultations^27^. Morbidity data (that is, symptoms and diseases) in this system are grouped under 19 main Read chapters. Data collected at the second hierarchical level or above were used to identify diagnostic groups. Individuals were defined as having osteoarthritis if they had consulted general practice for osteoarthritis between 2000 and 2008 based on Read code N05^27^ indicating a consultation for osteoarthritis. Survey age (date of birth) and sex data was used to check the correct person responded through matching with medical records by survey administrators and checked by RW.

#### ELSA subpopulation identifiers

Sex (male or female) was identified by self-report. Occupation type was identified from the earliest response to ELSA’s National Statistics Socio-economic Classification survey items about current (or recent or upcoming) main occupation. Occupation was not recorded at wave 1 and was unknown for individuals who did not respond to follow-up interviews. Occupation type was available in ELSA as job role type and categorised as non-manual (employers in large organisations, higher managerial occupations, higher professional occupations, lower professional and higher technical, lower managerial occupations, higher supervisory occupations, intermediate, employers in small organisations), manual (lower supervisory occupations, lower technical, semi-routine, routine), or self-employed (own account workers) or unknown (missing)^21,28^. ELSA variables were identified from survey responses and coded by the ELSA team.

#### Statistical methods

**Figure 1:**
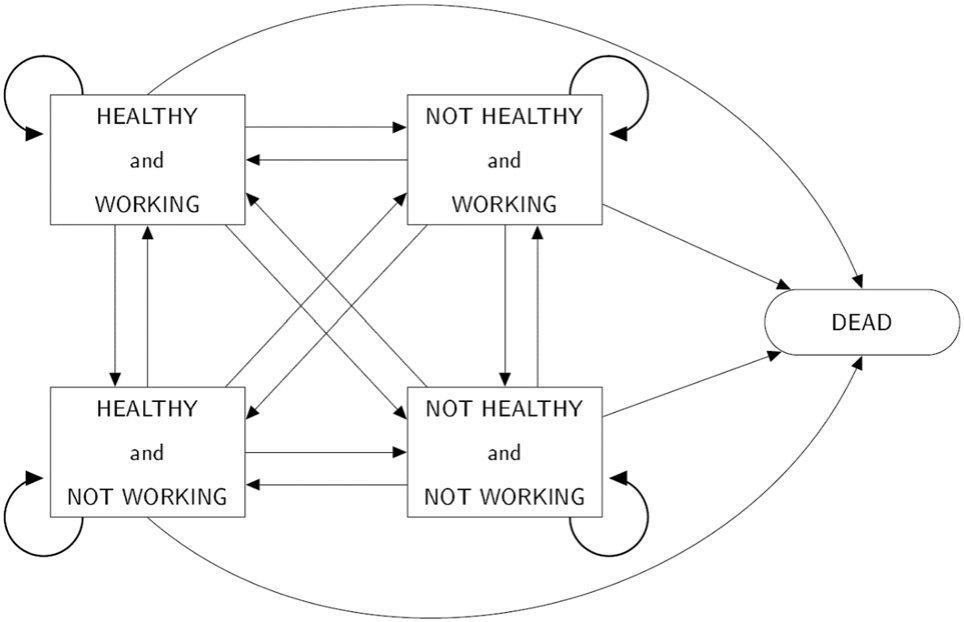
Healthy Working Life Expectancy multi-state model (permitted transitions shown with arrows). Reproduced from Parker et al.^3^.

At each assessment point respondents in both studies were classified into one of four alive health and work states (healthy and in work, healthy and not in work, not healthy and in work, and not healthy and not in work; Fig. 1) or dead (if so in future waves).

HWLE (and health expectancies for all other health and work states) was estimated with interpolated Markov chain multi-state modelling of cross-longitudinal survey data (panel data from repeated cross-sectional surveys of a cohort). A multi-state model was defined (Fig. 1). Interpolated Markov Chain software, IMaCh version 0.99r19, was used to estimate HWLE with standard errors^29^. This approach uses multinomial logistic regression to model the probabilities of transition from and to each HWLE state or to death over small discrete time intervals (interpolation steps) based on the transitions observed in the data, where the analysed time intervals are typically briefer than the time between data time points (here, interpolation step length was 12 months^3^). Maximum likelihood estimates of transition probability model parameters were found by evaluating the product of the transition probabilities for each step contained within each observed transition (health and work states at consecutive observed time points) or sequence of transitions (where more than one transition was observed for an individual). HWLE was estimated according to the health and work state occupied at age 50 years (and 65 years) and averaged (weighted by the observed prevalence of occupying each health and work state at age 50 and 65 years) to estimate HWLE for the population. Further details of the methodology and an investigation into changes in HWLE over time are provided elsewhere^3,29^. The approach is well-suited to longitudinal data from participants who enter and exit the study at different times or who have gaps of different lengths between observations. Thus, health expectancies (which indicate the average time spent in the healthy working state among people in a population) are not based on the assumption that a single point in time reflects the experiences of real people throughout the life course. Instead, health expectancies are inferred from data that follow individuals of various ages throughout portions their lives. Life expectancy (LE) was period life expectancy for the study period and was calculated as the sum of the four health expectancies.

Age was included in the transition probability models. In ELSA, age was measured in years from the midpoint of year of birth (taken as month 6; June) to the month and year of death. Age was exact in NorStOP. Transition probabilities were assumed to be constant over time within each year of age and not to be affected by any history of previous state occupation (the Markov property). Markov models enable and are well-suited to the study of health expectancy wherein individuals may move in and out of states in a defined state space^30,31^.

In ELSA, as osteoarthritis status could change over time, HWLE was estimated for people with and without osteoarthritis at age 50 (and for each year of age from 50 to 75) by including osteoarthritis as a covariate in the transition probability model. HWLE was also estimated for males and females as well as people with non-manual, manual, or self-employed occupation types by including variables sex and occupation type (using three dummy variables) in transition probability models separately and together with osteoarthritis. Individual observations were ignored if the participant had incomplete covariate data (OA, sex, occupation type) at that survey wave either due to non-response or incomplete survey response. Where covariate data were complete but health or work status was missing, the observation contributed to estimation of the probability of staying alive but not transitions between health and work states. All wave 1 respondents who did not survive to be interviewed again at a later wave had unknown occupation measurement because occupation type was not measured at wave 1. Therefore, for models including the occupation type, HWLE could not be calculated for the group with unknown occupation type as the statistical method relies on observed transitions between health and work states in the model state space. Further, this introduced selection bias to analyses including occupation type (but not other analyses) because transitions toward mortality were underrepresented (all wave 1 respondents with known occupation type survived at least until the next survey wave). HWLE as a percentage of LE was not calculated for manual, non-manual or self-employed occupation types due to this expected survivorship bias. Participants with unknown occupation remained in the analysis sample and contributed to estimation of the models (from which health expectancies were calculated).

In NorStOP, HWLE for people with and without osteoarthritis was investigated by stratifying the data according to osteoarthritis status, which was a fixed variable in the study. Due to the computationally intensity of IMaCh analyses, NorStOP’s fewer questionnaire time-points and less frequent data collection schedule compared to ELSA precluded the addition of osteoarthritis as a covariate in the model or the simultaneous investigation of further variables.

#### Sensitivity analyses

Previously published ELSA analyses suggested that health expectancies were robust to the use of yearly transition probability models (interpolation step size 12 months) compared to more computationally intensive monthly transition probability models (interpolation step size 1 month) in this dataset^3^. As occupation type was not measured in ELSA wave 1, resulting HWLE estimates will be compared to those previously published based on waves 2–6 only^3^.

As NorStOP had fewer assessment points than ELSA, the sensitivity of HWLE estimates to length of interpolation step size was investigated for this dataset by estimating monthly transition probability models for the NorStOP overall study population (not stratified by osteoarthritis). A further sensitivity analysis estimated HWLE for the NorStOP overall study population additionally including NorStOP participants without linked medical records. For comparison with the LE estimates from age 50 found by summing the health expectancies from each of the NorStOP analyses, total life expectancies from age 50 were additionally estimated with the life table method (observing mortality in the population without classifying individuals into health or work states).

### Role of the funding source

The funder had no involvement in any aspect of the study.

### Ethical approval

ELSA has received ethical approval from the South Central-Berkshire Research Ethics Committee. ELSA respondents gave their informed consent to participate in the study and for mortality data linkage. Ethical approval for all phases of NorStOP was obtained from the North Staffordshire Local Research Ethics Committee. A completed returned NorStOP questionnaire provided informed consent for inclusion. Medical record data were linked for NorStOP participants who gave informed consent to access medical records. All research was performed in accordance with relevant guidelines/regulations.

## Results

### HWLE for England

The ELSA study sample comprised 15,284 respondents (8259 women and 7025 men). The majority of study participants contributed at least two interviews (n = 12,232, 80.03%) and 2667 individuals were recorded to have died throughout the study period. There were 4659 participants (30.48%) who responded at all six waves. At each wave, the number of participants who responded and had complete health, work, and osteoarthritis data was: 11,170 (wave 1); 8658 (wave 2); 8562 (wave 3); 9593 (wave 4); 8868 (wave 5); 8735 (wave 6). The oldest age at time of response at any wave was 102.

**Figure 2:**
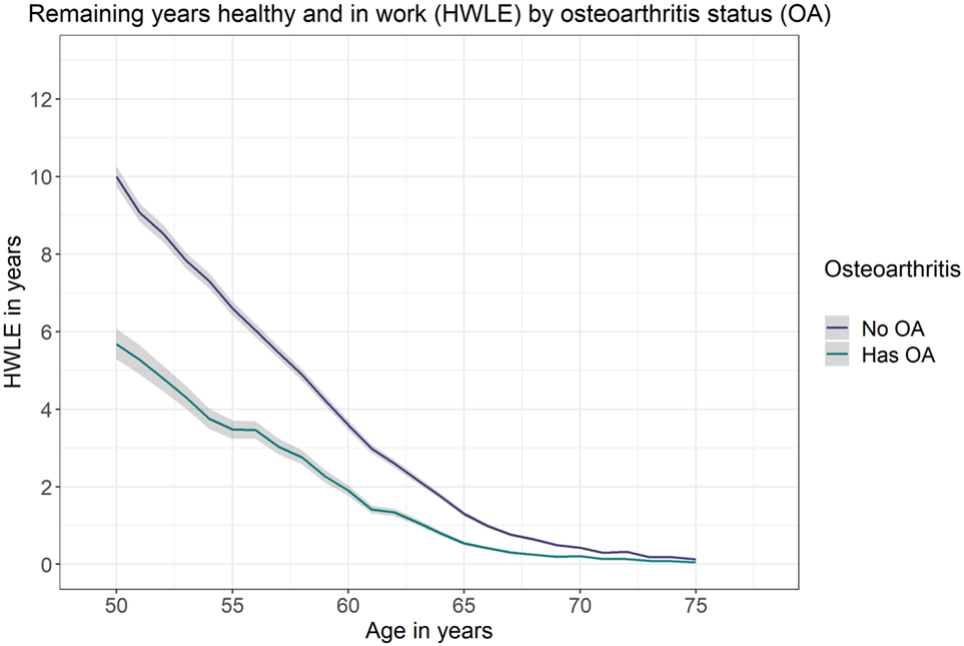
Plot of remaining years expected to be spent healthy and in work (HWLE) by osteoarthritis (OA) status at ages (ELSA data, n = 15,284). Osteoarthritis status is indicated by colour (no OA: purple; has OA: green).

**Figure 3:**
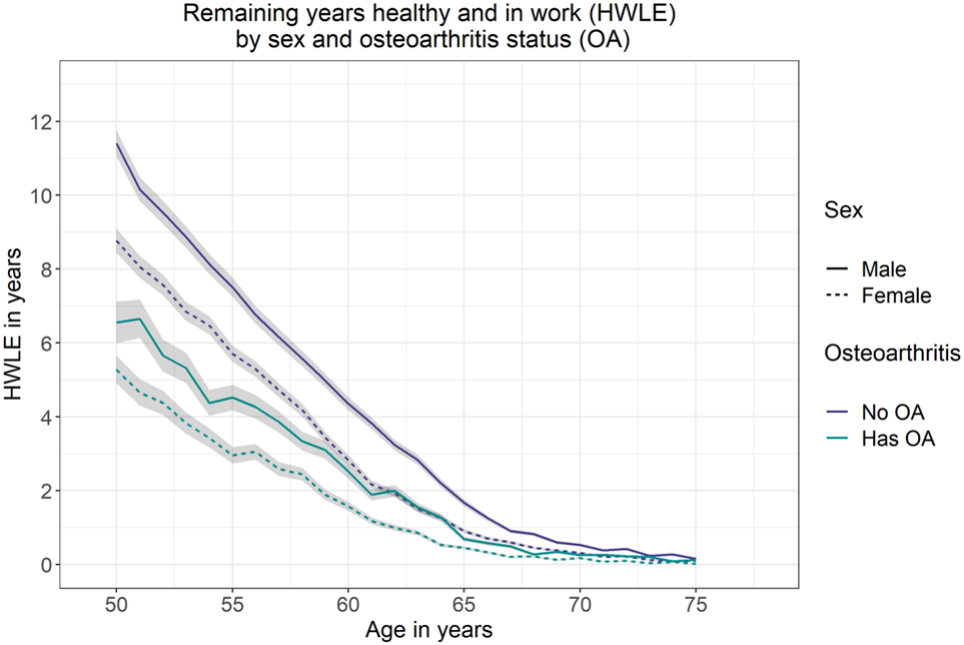
Plot of remaining years expected to be spent healthy and in work (HWLE) by sex and osteoarthritis (OA) status at ages 50–75 with 95% confidence intervals (ELSA data). Sex is indicated by line type (male: solid line; female: dashed line) and osteoarthritis status is indicated by colour (no OA: purple; has OA: green).

Estimates of HWLE and other health expectancies for England are presented in Table 1. At age 50, HWLE for England overall was 9.43 (95% confidence interval [9.19, 9.66]) years and life expectancy at age 50 was 31.83 (31.47, 32.18) years (Table 1). For people without osteoarthritis at age 50 in England, HWLE was 10.00 (9.74, 10.26) years, corresponding to 31.9% of LE from age 50 (31.31 [30.89, 31.73] years) (Fig. 2, Table 1). For people with osteoarthritis at age 50 in England, HWLE was 5.68 (5.29, 6.07) years, corresponding to 17.6% of LE from age 50 (32.25 [31.59, 32.90] years) (Fig. 2, Table 1). Both HWLE estimates were higher for men (with osteoarthritis: 6.56 [5.99, 7.12] years which was 21.6% of LE; without osteoarthritis: 11.41 [11.06, 11.75] years which was 37.9% of LE) and lower for women (with osteoarthritis: 5.28 [4.90, 5.65] years which was 15.7% of LE; without osteoarthritis: 8.77 [8.45, 9.09] years which was 26.7% of LE). This ordering was similar for HWLE at all ages from 50–75 (Fig. 3, Table 1).

**Table 1:** Health expectancies and life expectancy from age 50 with covariate combinations sex, osteoarthritis (OA) and occupation in England (ELSA data, n = 15,284).

Model	HWLE	State 2 (H&NW)	State 3 (NH&W)	State 4 (NH&NW)	LE
All participants	9.43 (9.19, 9.66)	11.24 (10.94, 11.53)	1.83 (1.73, 1.93)	9.33 (9.07, 9.59)	31.83 (31.47, 32.18)
Sex					
Male	11.00 (10.67, 11.34)	9.73 (9.34, 10.13)	1.99 (1.84, 2.15)	7.59 (7.26, 7.92)	30.32 (29.84, 30.8)
Female	8.19 (7.90, 8.48)	12.54 (12.12, 12.96)	1.69 (1.56, 1.82)	10.91 (10.52, 11.30)	33.33 (32.83, 33.84)
Osteoarthritis					
Has OA	5.68 (5.29, 6.07)	8.82 (8.36, 9.29)	2.74 (2.48, 3.00)	15.00 (14.43, 15.58)	32.25 (31.59, 32.90)
No OA	10 (9.74, 10.26)	12.17 (11.8, 12.53)	1.62 (1.51, 1.72)	7.53 (7.25, 7.80)	31.31 (30.89, 31.73)
Occupation					
Self-employed	10.98 (10.23, 11.74)	10.70 (9.58, 11.82)	2.78 (2.37, 3.19)	8.99 (7.94, 10.05)	33.45 (31.89, 35.02)
Non-manual	10.06 (9.74, 10.38)	14.32 (13.78, 14.85)	1.79 (1.65, 1.93)	10.27 (9.80, 10.75)	36.44 (35.74, 37.14)
Manual	8.59 (8.24, 8.93)	11.40 (10.93, 11.88)	1.76 (1.61, 1.92)	12.05 (11.55, 12.55)	33.80 (33.16, 34.45)
Sex + osteoarthritis					
Males					
Has OA	6.56 (5.99, 7.12)	7.36 (6.83, 7.88)	3.73 (3.33, 4.12)	12.75 (12.06, 13.44)	30.39 (29.6, 31.17)
No OA	11.41 (11.06, 11.75)	10.41 (9.97, 10.84)	1.78 (1.64, 1.93)	6.47 (6.16, 6.79)	30.07 (29.55, 30.59)
Females					
Has OA	5.28 (4.90, 5.65)	9.65 (9.12, 10.18)	2.38 (2.12, 2.64)	16.23 (15.58, 16.88)	33.54 (32.81, 34.26)
No OA	8.77 (8.45, 9.09)	13.96 (13.44, 14.47)	1.46 (1.33, 1.59)	8.66 (8.27, 9.05)	32.85 (32.27, 33.42)
Osteoarthritis + occupation					
People with OA					
Self employed	5.81 (4.86, 6.76)	8.21 (7.10, 9.31)	5.16 (4.35, 5.97)	14.28 (12.72, 15.85)	33.46 (31.60, 35.31)
Non-manual	7.29 (6.80, 7.78)	11.10 (10.45, 11.76)	2.93 (2.61, 3.25)	15.02 (14.26, 15.78)	36.34 (35.41, 37.26)
Manual	4.86 (4.46, 5.25)	8.61 (8.05, 9.17)	2.19 (1.92, 2.46)	17.98 (17.18, 18.78)	33.63 (32.73, 34.53)
People without OA					
Self employed	12.00 (11.23, 12.78)	11.62 (10.40, 12.83)	2.32 (1.95, 2.68)	7.36 (6.44, 8.28)	33.29 (31.71, 34.88)
Non-manual	10.48 (10.14, 10.82)	15.75 (15.11, 16.38)	1.58 (1.45, 1.72)	8.42 (7.96, 8.88)	36.23 (35.47, 36.99)
Manual	9.33 (8.94, 9.71)	12.68 (12.12, 13.24)	1.58 (1.43, 1.73)	9.91 (9.42, 10.40)	33.50 (32.79, 34.20)
Sex + osteoarthritis + occupation					
Males with OA					
Self employed	7.80 (6.57, 9.04)	6.91 (5.94, 7.87)	6.46 (5.46, 7.46)	11.22 (9.78, 12.65)	32.38 (30.68, 34.09)
Non-manual	10.30 (9.62, 10.98)	9.31 (8.62, 10.00)	2.92 (2.50, 3.35)	11.67 (10.90, 12.44)	34.20 (33.26, 35.15)
Manual	2.01 (1.57, 2.46)	7.27 (6.58, 7.95)	1.32 (0.94, 1.71)	19.00 (17.98, 20.01)	29.60 (28.48, 30.71)
Males without OA					
Self employed	13.06 (12.27, 13.86)	10.27 (9.13, 11.40)	2.62 (2.20, 3.04)	6.39 (5.56, 7.23)	32.35 (30.81, 33.88)
Non-manual	12.06 (11.63, 12.49)	13.55 (12.89, 14.22)	1.64 (1.47, 1.81)	7.19 (6.73, 7.65)	34.44 (33.66, 35.23)
Manual	10.22 (9.75, 10.68)	10.73 (10.14, 11.32)	1.76 (1.56, 1.95)	8.82 (8.30, 9.34)	31.52 (30.77, 32.27)
Females with OA					
Self employed	2.99 (2.26, 3.71)	10.11 (8.65, 11.57)	2.60 (1.66, 3.54)	19.50 (17.52, 21.49)	35.20 (33.05, 37.34)
Non-manual	6.34 (5.86, 6.82)	12.19 (11.45, 12.93)	2.86 (2.53, 3.19)	16.73 (15.87, 17.59)	38.11 (37.09, 39.13)
Manual	4.95 (4.54, 5.36)	9.39 (8.77, 10.01)	2.22 (1.95, 2.50)	18.98 (18.10, 19.86)	35.54 (34.57, 36.52)
Females without OA					
Self employed	10.25 (9.47, 11.02)	14.55 (13.04, 16.07)	1.82 (1.45, 2.18)	9.49 (8.35, 10.64)	36.11 (34.30, 37.92)
Non-manual	9.04 (8.65, 9.43)	18.12 (17.32, 18.93)	1.50 (1.35, 1.65)	9.82 (9.22, 10.41)	38.48 (37.55, 39.41)
Manual	8.57 (8.15, 8.99)	14.47 (13.77, 15.17)	1.47 (1.31, 1.63)	11.17 (10.55, 11.79)	35.67 (34.82, 36.53)

Results shown are mean average duration in the given health/work state in years with 95% confidence interval.

*HWLE* healthy working life expectancy, *H&NW* Healthy and Not Working, *NH&W* Not Healthy and Working, *NH&NW* Not Healthy and Not Working, *LE* life expectancy.

**Figure 4:**
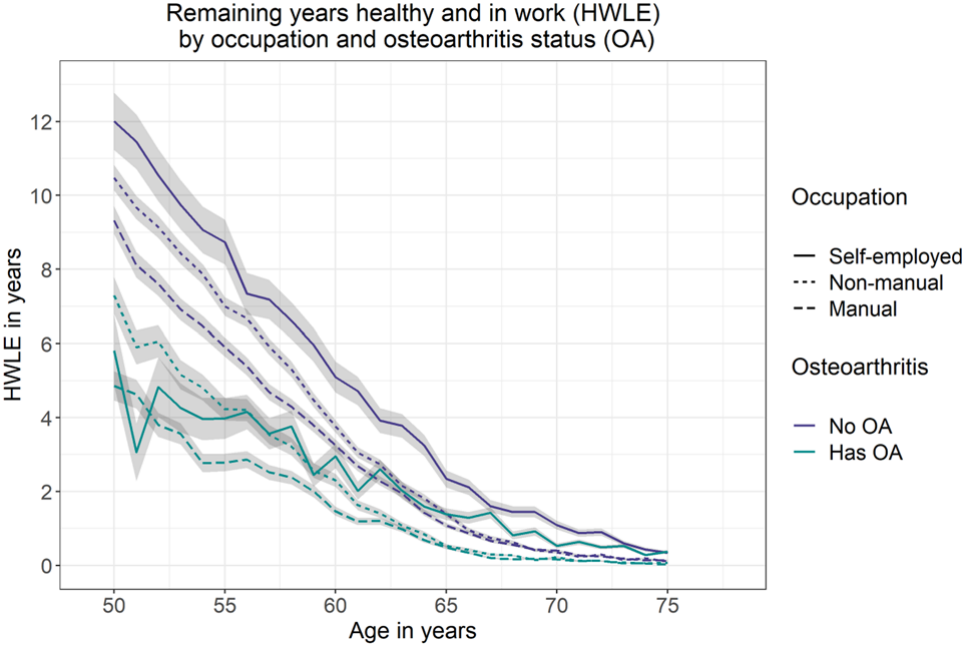
Plot of remaining years expected to be spent healthy and in work (HWLE) by occupation and osteoarthritis (OA) status at ages 50–75 with 95% confidence intervals (ELSA data, n = 15,284). Occupation type is indicated by line type (self-employed: solid line; non-manual: short dashed line; manual: long dashed line) and osteoarthritis status is indicated by colour (no OA: purple; has OA: green).

**Figure 5:**
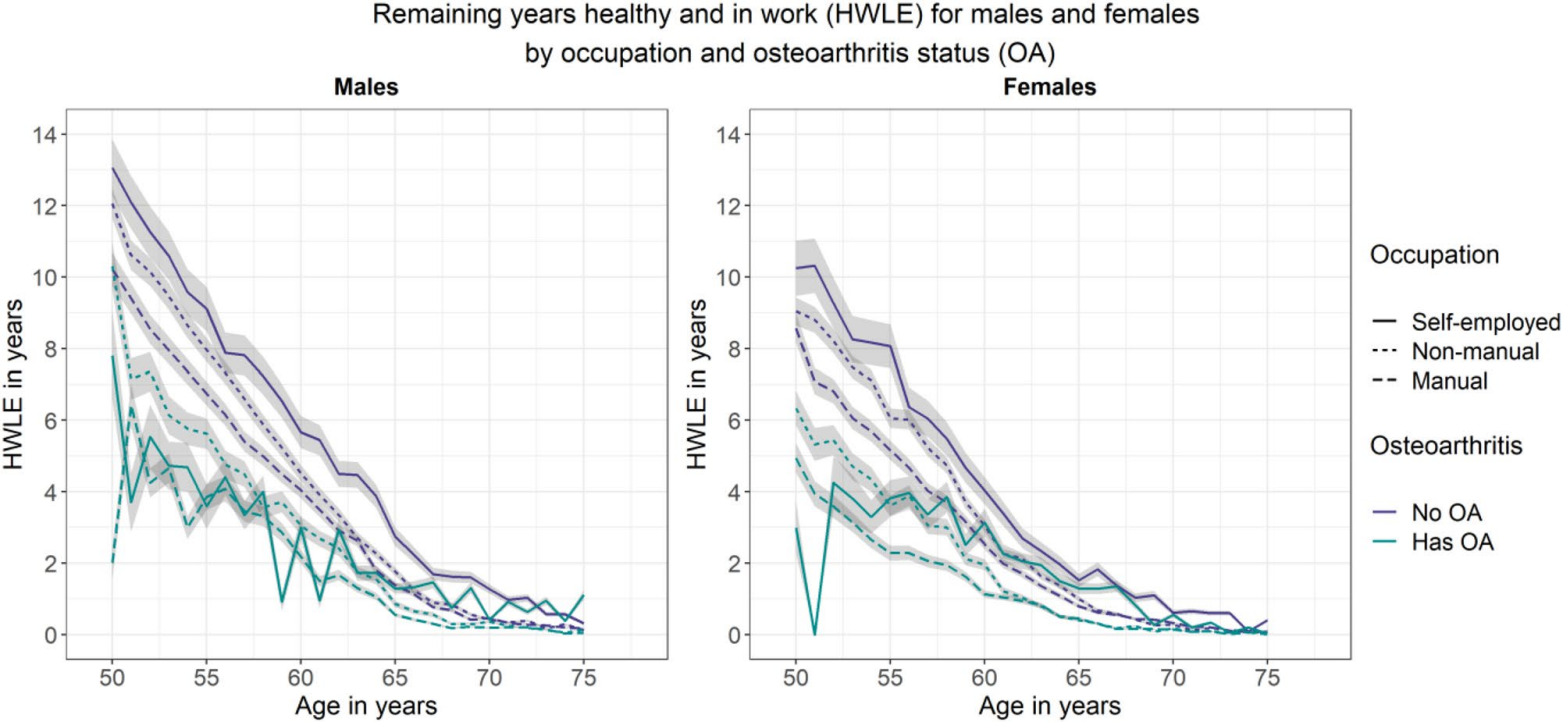
Plot of remaining years expected to be spent healthy and in work (HWLE) by sex, occupation, and osteoarthritis (OA) status at ages 50–75 with 95% confidence intervals (ELSA data, n = 15,284). Occupation type is indicated by line type (self-employed: solid line; non-manual: short dashed line; manual: long dashed line) and osteoarthritis status is indicated by colour (no OA: purple; has OA: green).

Analyses of HWLE by occupation type showed that, for England overall as well as for people with osteoarthritis and people without osteoarthritis, HWLE was highest for people in self-employed occupations followed by non-manual occupations and lowest for people in manual occupations (Fig. 4, Table 1). This ordering persisted among men, among women, among those with osteoarthritis, and among those without osteoarthritis—with the exception that HWLE estimates for self-employed people with osteoarthritis fluctuated around other estimates at younger ages suggesting insufficient data due to low numbers of self-employed people with osteoarthritis aged 50–60 (Fig. 5, Table 1).

At age 65, remaining HWLE was 1.30 (1.24, 1.37) years for people without osteoarthritis and 0.54 (0.49, 0.58) years for people with osteoarthritis (Fig. 2, Table 2). On average, 65-year-old women in England can expect to spend 0.90 (0.84, 0.96) years healthy and working if they do not have osteoarthritis, or 0.45 (0.41, 0.49) years healthy and working if they do have osteoarthritis (Fig. 3, Table 2). On average, 65-year-old men in England can expect to spend 1.67 (1.58, 1.76) years healthy and working if they do not have osteoarthritis, or 0.69 (0.63, 0.76) years healthy and working if they do have osteoarthritis (Fig. 3, Table 2).

**Table 2:** Remaining years expected to be spent healthy and in work (HWLE) at ages 50, 55, 60, 65, 70 and 75 with covariate combinations sex, osteoarthritis (OA) and occupation in England (ELSA data, n = 15,284).

Model	Age (95% CI)					
	50	55	60	65	70	75
Sex						
Male	11.00 (10.67, 11.34)	7.06 (6.83, 7.30)	3.96 (3.80, 4.11)	1.50 (1.42, 1.58)	0.45 (0.41, 0.48)	0.15 (0.14, 0.16)
Female	8.19 (7.90, 8.48)	5.08 (4.90, 5.27)	2.50 (2.39, 2.60)	0.75 (0.70, 0.80)	0.25 (0.23, 0.27)	0.06 (0.05, 0.07)
Osteoarthritis						
Has OA	5.68 (5.29, 6.07)	3.48 (3.24, 3.72)	1.91 (1.77, 2.04)	0.54 (0.49, 0.58)	0.21 (0.19, 0.23)	0.05 (0.05, 0.06)
No OA	10.00 (9.74, 10.26)	6.60 (6.42, 6.78)	3.59 (3.47, 3.71)	1.30 (1.24, 1.37)	0.43 (0.40, 0.46)	0.13 (0.12, 0.14)
Occupation						
Self-employed	10.98 (10.23, 11.74)	7.92 (7.36, 8.49)	4.61 (4.24, 4.99)	2.14 (1.94, 2.34)	0.94 (0.84, 1.03)	0.35 (0.31, 0.39)
Non-manual	10.06 (9.74, 10.38)	6.51 (6.29, 6.73)	3.41 (3.27, 3.55)	1.16 (1.09, 1.22)	0.30 (0.28, 0.33)	0.09 (0.08, 0.10)
Manual	8.59 (8.24, 8.93)	5.15 (4.93, 5.37)	2.77 (2.64, 2.90)	0.92 (0.86, 0.98)	0.30 (0.28, 0.33)	0.10 (0.09, 0.10)
Sex + osteoarthritis						
Males						
Has OA	6.56 (5.99, 7.12)	4.52 (4.18, 4.86)	2.52 (2.33, 2.72)	0.69 (0.63, 0.76)	0.26 (0.23, 0.28)	0.13 (0.11, 0.14)
No OA	11.41 (11.06, 11.75)	7.51 (7.25, 7.76)	4.36 (4.18, 4.53)	1.67 (1.58, 1.76)	0.53 (0.49, 0.57)	0.16 (0.14, 0.17)
Females						
Has OA	5.28 (4.90, 5.65)	2.96 (2.74, 3.18)	1.58 (1.46, 1.69)	0.45 (0.41, 0.49)	0.18 (0.16, 0.20)	0.02 (0.02, 0.03)
No OA	8.77 (8.45, 9.09)	5.70 (5.48, 5.92)	2.83 (2.70, 2.96)	0.90 (0.84, 0.96)	0.31 (0.28, 0.34)	0.09 (0.08, 0.10)
Osteoarthritis + occupation						
People with OA						
Self employed	5.81 (4.86, 6.76)	3.98 (3.43, 4.54)	2.96 (2.63, 3.29)	1.39 (1.23, 1.55)	0.53 (0.46, 0.59)	0.38 (0.33, 0.42)
Non-manual	7.29 (6.80, 7.78)	4.23 (3.92, 4.54)	2.30 (2.13, 2.48)	0.53 (0.48, 0.58)	0.23 (0.21, 0.25)	0.07 (0.06, 0.07)
Manual	4.86 (4.46, 5.25)	2.79 (2.55, 3.02)	1.47 (1.35, 1.59)	0.49 (0.44, 0.54)	0.17 (0.15, 0.18)	0.03 (0.02, 0.03)
People without OA						
Self employed	12.00 (11.23, 12.78)	8.74 (8.13, 9.34)	5.10 (4.68, 5.51)	2.34 (2.11, 2.57)	1.09 (0.97, 1.21)	0.35 (0.30, 0.39)
Non-manual	10.48 (10.14, 10.82)	6.99 (6.74, 7.24)	3.77 (3.60, 3.93)	1.39 (1.31, 1.48)	0.36 (0.32, 0.39)	0.10 (0.09, 0.12)
Manual	9.33 (8.94, 9.71)	5.90 (5.64, 6.16)	3.25 (3.08, 3.41)	1.08 (1.01, 1.15)	0.40 (0.37, 0.44)	0.14 (0.13, 0.15)
Sex + osteoarthritis + occupation						
Males with OA						
Self employed	7.80 (6.57, 9.04)	3.59 (2.97, 4.21)	3.00 (2.66, 3.34)	1.29 (1.14, 1.43)	0.42 (0.35, 0.48)	1.11 (0.98, 1.24)
Non-manual	10.30 (9.62, 10.98)	5.63 (5.20, 6.05)	3.04 (2.78, 3.30)	0.86 (0.76, 0.95)	0.36 (0.32, 0.40)	0.14 (0.13, 0.16)
Manual	2.01 (1.57, 2.46)	3.85 (3.49, 4.22)	2.18 (1.98, 2.37)	0.56 (0.49, 0.62)	0.19 (0.16, 0.21)	0.06 (0.05, 0.06)
Males without OA						
Self employed	13.06 (12.27, 13.86)	9.11 (8.49, 9.72)	5.67 (5.22, 6.12)	2.74 (2.48, 3.00)	1.28 (1.14, 1.41)	0.31 (0.26, 0.36)
Non-manual	12.06 (11.63, 12.49)	7.96 (7.63, 8.30)	4.51 (4.28, 4.74)	1.77 (1.65, 1.88)	0.42 (0.38, 0.46)	0.13 (0.11, 0.14)
Manual	10.22 (9.75, 10.68)	6.74 (6.41, 7.07)	4.05 (3.82, 4.27)	1.38 (1.28, 1.48)	0.46 (0.42, 0.51)	0.18 (0.16, 0.20)
Females with OA						
Self employed	2.99 (2.26, 3.71)	3.82 (3.30, 4.34)	3.13 (2.75, 3.52)	1.30 (1.14, 1.45)	0.55 (0.48, 0.63)	0.03 (0.01, 0.04)
Non-manual	6.34 (5.86, 6.82)	3.61 (3.31, 3.90)	1.96 (1.80, 2.11)	0.41 (0.36, 0.45)	0.18 (0.16, 0.19)	0.04 (0.03, 0.04)
Manual	4.95 (4.54, 5.36)	2.28 (2.07, 2.49)	1.13 (1.03, 1.23)	0.45 (0.41, 0.50)	0.15 (0.13, 0.16)	0.01 (0.01, 0.01)
Females without OA						
Self employed	10.25 (9.47, 11.02)	8.07 (7.45, 8.68)	4.03 (3.65, 4.41)	1.52 (1.33, 1.71)	0.61 (0.53, 0.69)	0.41 (0.35, 0.46)
Non-manual	9.04 (8.65, 9.43)	6.06 (5.79, 6.34)	3.05 (2.88, 3.22)	1.00 (0.92, 1.07)	0.28 (0.25, 0.31)	0.07 (0.06, 0.08)
Manual	8.57 (8.15, 8.99)	5.16 (4.89, 5.43)	2.54 (2.38, 2.69)	0.80 (0.73, 0.86)	0.33 (0.30, 0.36)	0.10 (0.08, 0.11)

Self-employed 65-year-olds in England can expect to spend an average of 2.34 (2.11, 2.57) years healthy and working if they do not have osteoarthritis, or 1.39 (1.23, 1.55) years if they do have osteoarthritis (Fig. 4, Table 2). At age 65, those with non-manual occupations can expect to spend an average of 1.39 (1.31, 1.48) years healthy and working if they do not have osteoarthritis, or 0.53 (0.48, 0.58) years if they do have osteoarthritis (Fig. 4, Table 2). Remaining HWLE at age 65 for those in manual occupations was 1.08 (1.01, 1.15) years for people with osteoarthritis compared to 0.49 (0.44, 0.54) years for people without osteoarthritis (Fig. 4, Table 2). ELSA sample sizes by region were insufficient to estimate HWLE according to osteoarthritis status for all regions (either by stratification or by inclusion of osteoarthritis as a covariate).

### HWLE for North Staffordshire

The NorStOP study sample comprised 13,774 respondents (7373 women and 6401 men) who had consented to share medical record data. The mean follow-up time was 3.21 years with 21.40% of participants providing response at two time points (baseline and one follow-up) and 42.77% of participants responding to all three surveys. The oldest age at time of response at any time point was 98.

HWLE at age 50 for the overall NorStOP (North Staffordshire) population was estimated as 6.58 (6.28, 6.87) years, which was 22.5% of LE from age 50 (Table 3). In addition to spending 6.58 years healthy and in work from age 50, on average people age 50 were expected to spend 6.96 (6.63, 7.30) years healthy and not in work, 3.32 (3.09, 3.54) years not healthy and in work, and 12.38 (11.95, 12.80) years not healthy and not in work (Appendix Table 1). Total LE was 29.23 (28.77, 29.69) years (Table 3, Appendix Table 1).

**Table 3:** HWLE and LE at ages 50 and 65 in North Staffordshire overall and subpopulations with and without osteoarthritis (OA) (NorStOP data, n = 13,774).

Population	Sample size	HWLE at age 50	LE at age 50	HWLE at age 65	LE at age 65
North Staffordshire	13,774	6.58 (6.28, 6.87)	29.23 (28.77, 29.69)	0.27 (0.24, 0.30)	15.99 (15.65, 16.33)
OA group	3260	4.31 (3.68, 4.94)	31.82 (30.98, 32.67)	0.19 (0.14, 0.25)	17.85 (17.19, 18.51)
Non-OA group	10,514	6.90 (6.57, 7.24)	28.39 (27.86, 28.92)	0.30 (0.26, 0.34)	15.32 (14.93, 15.72)

HWLE for the osteoarthritis group was 4.31 (3.68, 4.94) years (13.5% of LE), approximately two years lower than the overall NorStOP population (Table 3). People with osteoarthritis were also expected to spend 5.35 (4.72, 5.98) years not healthy and in work, 3.47 (2.94, 4.01) years not healthy and in work, and 18.69 (17.65, 19.72) years not healthy and not in work from age 50 (Appendix Table 1). Total LE from age 50 for people who consulted primary care for osteoarthritis was 31.82 (30.98, 32.67) years (Table 3). Compared to those with osteoarthritis, the non-osteoarthritis group had longer HWLE (6.90 [6.57, 7.24] years, 24.3% of LE), spent more years healthy and not in work (7.43 [7.04, 7.83] years), and fewer years not heathy and in work (3.22 [2.98, 3.47] years) and not healthy and not in work (10.83 [10.38, 11.28] years) from age 50 (Table 3, Appendix Table 1). HWLE was similar (point estimate was slightly higher) for people without osteoarthritis compared to the overall NorStOP population (Table 3). LE from age 50 for the non-osteoarthritis group was 28.39 (27.86, 28.92) years (Table 3).

HWLE at age 65 for the overall NorStOP study population was 0.27 years (0.24, 0.30) (Table 3). HWLE at age 65 was 0.19 years (0.14, 0.25) for the osteoarthritis group and 0.30 years (0.26, 0.34) for the non-osteoarthritis group (Table 3).

### ELSA sensitivity analyses

As expected, the estimates of total LE according to occupation type (Table 1) were higher than those previously published based on ELSA waves 2–6 (excluding wave 1): 34.54 (33.92, 35.16) years (non-manual), 31.66 (31.01, 32.31) years (manual), and 31.64 (29.95, 33.33) years (self-employed)^3^. That LE (which is calculated as is the sum of the four health expectancies) appears to be overestimated implies that at least one health expectancy is overestimated (and it is possible that others might be underestimated) using the models including occupation type. LE estimates were also high in models including occupation type and other covariate(s). Despite higher estimates of LE, estimates of HWLE at age 50 for manual and non-manual occupation types were similar but slightly lower than those previously published based on ELSA waves 2–6: 10.06 (9.74, 10.38) years for people with non-manual occupations compared to 10.32 (9.95, 10.69) years previously estimated; and 8.59 (8.24, 8.93) years for people with manual occupations compared to 8.72 (8.25, 9.20) years previously estimated. HWLE was estimated for people with self-employed occupations as 10.98 (10.23, 11.74) years from age 50 in this study of ELSA waves 1–6, compared to 11.76 (10.76, 12.76) years previously estimated from ELSA waves 2–6.

### NorStOP sensitivity analyses

Health expectancy results estimates from the main NorStOP study sample were similar (with slightly higher point estimates) to estimates found when including people with missing medical record data (Appendix Table 2). Total LE from the main study sample using the life table method was lower than that found by summing the health expectancies and was closer to estimates found using the larger sample (Appendix Table 2). HWLE point estimates were higher when estimated using monthly transition probability models than yearly transition probability models in both the main study sample (6.90 [6.12, 7.68] years compared to 6.58 [6.28, 6.87] years) and the larger sample including people with missing medical record data (6.93 [6.31, 7.55] compared to 6.52 [6.24, 6.80]) (Appendix Table 2). Health expectancy estimates for the NorStOP population subgroup with osteoarthritis were based on fewer observed transitions than estimates for the subgroup without osteoarthritis. LE estimates from summed health expectancies for population subgroups with and without osteoarthritis were similar to estimates found using the life table method (Appendix Table 2). The estimate of total LE for the osteoarthritis group was found to be higher than for people without osteoarthritis and higher than the general population estimate (Table 3, Appendix Table 2).

## Discussion

The analyses presented in this study of HWLE in England and in the local area North Staffordshire indicate that length of healthy working life from age 50 is a third less for people with osteoarthritis than people without osteoarthritis. In England on average, people who have osteoarthritis by age 50 are expected to spend fewer than six years of their remaining life expectancy both healthy and in work—over four years fewer than those without osteoarthritis. HWLE for people with osteoarthritis in North Staffordshire is 4.31 years, over a year lower than the England average. Within groupings by occupation and sex, people with osteoarthritis have lower HWLEs than their counterparts without osteoarthritis. The association of osteoarthritis with lower HWLE can be seen at all years of age from 50 to 77. Although a higher number of people may have osteoarthritis and lower HWLE at older years in the age range, the inequality at age 50 highlights the impact of osteoarthritis on the ability of individuals work until they are older before reaching State Pension age. These findings call into question the feasibility of policies to extend working lives as osteoarthritis affects over one in four older workers^11,12^. Furthermore, estimates of LE and HWLE at age 50 were over three years lower in the NorStOP population than in England on average, which suggests that differences in health outcomes and employment factors (including work opportunities) affect HWLE in local areas to an even greater extent than implied by the regional variation observed previously (NorStOP HWLE: 6.58 [6.28, 6.87] years; ELSA HWLE: 9.43 [9.19, 9.66] years)^3^.

Lower HWLE for people with osteoarthritis highlights the detrimental effect associated with this common disease on people’s access to or ability to participate in paid work. The smaller difference in HWLE between the osteoarthritis and non-osteoarthritis groups in NorStOP compared to ELSA suggests that the impact of osteoarthritis on HWLE in a deprived area is less because the average length of healthy working life is already lower. This highlights the importance of upstream factors and deprivation; health conditions such as osteoarthritis may be associated with lower HWLE, but broader social factors also influence health and work outcomes. Using ELSA data, the higher estimates of HWLE for self-employed people compared to those of other occupation types indicates higher levels of functional health; people in self-employed work may often have more control over their activities and therefore be better placed to manage their health conditions more effectively (which could also be a reason for choosing self-employment).

Lower HWLE may be linked to osteoarthritis symptoms of pain and reduced physical function limitation, which may become worse with time and age. These symptoms are likely to be influenced by various lifestyle, socioeconomic, workplace, and social factors^31–33^. Health-related quality of life is closely related to pain severity, and pain interference is strongly associated with work disability and premature work loss^34,35^. However, supportive workplaces can improve return to work, sickness absence, and presenteeism outcomes for people with chronic conditions^32,36^. This is consistent with the findings of this study: models that included osteoarthritis and occupation type suggested that, by age 60, people with osteoarthritis who are self-employed achieve similar HWLE outcomes as people without osteoarthritis in non-manual or manual occupations. This highlights the importance of measuring HWLE as an indicator as other factors (not only medical diagnoses) from the wider biopsychosocial model of health and work can also drive ability to engage in paid work. Comorbidities with osteoarthritis are also prevalent and may contribute to the association observed with lower HWLE.

### Strengths and limitations

The presentation of HWLE results from nationally (ELSA) and locally (NorStOP) representative study populations is a strength of this work as it allows the identification of differences in HWLE at a local/regional level. While ELSA data may underrepresent people living in more deprived areas, the use of NorStOP also allows estimation of HWLE in a local population which on average is more deprived than other areas of England; North Staffordshire includes several of England’s most deprived local areas^37^. The consistency of LE estimates from summed health expectancies with LE from the life table method across all ELSA analyses and NorStOP HWLE analyses indicates sufficient sample sizes and suggests that HWLE estimates are a realistic representation of the average health and work outcomes experienced by the study participants.

ELSA HWLE and LE results in this study for England overall and for men and women separately were similar to those published previously using data from the same ELSA participants but which did not require handling of covariate missing data^3^. However, self-report of doctor diagnosed osteoarthritis may have been a limitation to the use of ELSA data due to inconsistencies in osteoarthritis reporting (a quarter of osteoarthritis reports in waves 1–5 were disputed at a later wave). Furthermore, some individuals with OA symptoms may be less likely to seek healthcare—for example, due to perceptions that treatment cannot help control symptoms or that the condition is an inevitable part of ageing^38^. It was assumed in this study that osteoarthritis is not reversible (that is, once structural change has occurred it will not reverse back to “normal” anatomy). Potential misclassification bias could lead to underestimation of the gap in HWLE between people with and without osteoarthritis.

In ELSA, the first available record of occupation type was used as an indicator of main employment type throughout the life course, but some participants may have changed job type (e.g. for health reasons). Survivorship bias also affected participants with known occupation type as this was only measured from wave 2. As expected, LE estimates by occupation type were higher than the overall ELSA LE and higher than occupation type subpopulation estimates using waves 2–6 only (to account for the survivorship bias) published previously^3^. However, there was no evidence that HWLE estimates were affected by this bias (likely due to low rates of transition to death directly from the healthy and working state, with healthy working years typically lived closer to age 50 than end of life). As health expectancies sum to LE, this implies overestimation of at least one other health expectancy.

An important limitation affecting more complex models used to analyse ELSA data is the presence of infrequently observed transitions particularly among participants with osteoarthritis (who comprised a smaller group than participants without osteoarthritis, especially at younger ages). This led to uncertain HWLE estimates without smooth trends across the years of age^3^.

Analysis of NorStOP data in this study allowed osteoarthritis and HWLE to be explored at local area level—a level of detail that is not currently feasible with national ELSA data. The comparability of ELSA and NorStOP results is limited by differences in study sampling frame, questions used to assess health, osteoarthritis definition, and method of estimating HWLE for subpopulations according to osteoarthritis status (ELSA analyses included osteoarthritis as a covariate while NorStOP data were stratified). However, although health definitions were different, the operationalisation of health in both datasets as self-reported long-standing limiting illness incorporates a subjective aspect that captures the biopsychosocial relationship between health and work (e.g. limitations experienced due to illness may sometimes be removed through employer adaptations, increased demand for labour, or effective condition management)^2,39^. Furthermore, assessing long-standing illness focuses on ability to function, which is an aspect of health that links more directly with work capacity than self-assessed health (which may be interpreted inconsistently between individuals and across cultures and is associated with respondents’ feelings of vitality)^40^. Both ELSA and NorStOP results were produced using the IMaCh approach, which was designed for generating theoretically and methodologically appropriate health expectancy estimates. The high data requirements for this method prevented examination of osteoarthritis together with region using ELSA data and may be a barrier to the sustainability of HWLE calculation using existing methods due to few ongoing longitudinal surveys with linked mortality data.

In both datasets, assessment of osteoarthritis was operationalised based on doctor diagnosis. NorStOP’s linked medical records also allowed more direct identification of doctor diagnosed osteoarthritis. A limitation of NorStOP data analyses was the exclusion of study participants without linked medical records. However, although LE estimates showed some sensitivity to this, results indicated that HWLE estimates were robust to the exclusion of participants without linked medical record data. Sensitivity analyses suggested some sensitivity of NorStOP data analyses to interpolation step size, which was not detected for ELSA analyses. The reason for this sensitivity is unclear but could be linked to the frequency of data collection (every three years in NorStOP and every two years in ELSA), sample size or characteristics, or the number or distribution of transitions.

The NorStOP osteoarthritis group LE from age 50 exceeded that of the non-osteoarthritis group, which is unlikely to reflect the true difference in the North Staffordshire population as an association between osteoarthritis and increased mortality has previously been found using data from the same study after adjusting for confounding^41^. The similarity of LE estimates between summed health expectancies estimated with the multi-state model and that estimated using the life table method implied that the health and work experiences recorded in the data for the study population were not over- or underestimated. The higher LE in the osteoarthritis group compared to the non-osteoarthritis group could be due to low numbers of questionnaires completed by the oldest old adults (≥ 85 years), especially among those without osteoarthritis, suggesting a need for more data collected this age group or longer follow-up for mortality records. If available mortality records predominantly relate to younger adults who had participated in the study, life expectancy estimates may be strongly influenced by a small number of (probably healthier than average) adults who are participating in the study at the oldest ages. Again, this is more likely to impact other health expectancies than HWLE as participants at oldest old ages are not likely to be both healthy (without long-standing limiting illness) and in paid employment.

That the ELSA and NorStOP data cover the same period is advantageous for comparison. However, although the North Staffordshire population has not changed in terms of deprivation and ethnicity composition since data collection, the national population has become more ethnically diverse and employment factors change over time^37,42,43^.

### Implications for policy and research

As the prevalence of osteoarthritis among workers increases (through population and workforce ageing, deferred retirement age, and increasing obesity and physical inactivity), the association between osteoarthritis and lower HWLE may make extensions to working life difficult for many people with this common musculoskeletal condition. However, findings such as longer healthy working lives for self-employed people caution against an interpretation of inevitability. An important implication for research is therefore the need to understand—in the general population and in particular among those with osteoarthritis—how biopsychosocial factors drive work participation and work outcomes such as absenteeism (work absence e.g. for health reasons) and presenteeism (reduced productivity while at work due to illness or for other reasons). Achieving extended working lives policies may require changing perceptions around work disability as a likely consequence of osteoarthritis and other musculoskeletal disorders. Further work is needed to determine the extent that interference of symptomatic osteoarthritis with everyday activities (such as work and social engagement) may be lessened with supportive workplaces and a higher degree of individual control of work responsibilities and arrangements. Proactive population/public health and primary care approaches targeting maintenance of workers’ health could prevent health conditions such as osteoarthritis impacting on work participation. Early identification (perhaps via presenteeism) and intervention could extend healthy working lives not just for workers with musculoskeletal conditions but also for those with other physical and mental health conditions.

Osteoarthritis is one example of various prevalent conditions that might impact on work in older workers. That national datasets may not have the sample size to examine regional variation in HWLE for specific health conditions suggests a role for local data (as demonstrated in this study using NorStOP) and methodological development (e.g. to allow greater use of national survey datasets) in order to identify ways to increase HWLE for these groups. A better understanding of regional variation in HWLE for subpopulations with osteoarthritis would help to inform national and local policy decisions and interventions to promote health and wellbeing as the State Pension age rises, which could avoid exacerbating existing challenges in areas with low employment and poor health outcomes.

## Conclusion

Evidence from two large longitudinal studies analysed in this study show that people with osteoarthritis are expected to spend fewer years healthy and in work from age 50 compared to people without osteoarthritis. The population subgroup identified by sex, osteoarthritis and occupation type that was best placed to work extended working lives were self-employed men without osteoarthritis—who were expected to be healthy and in work for just over 13 years from age 50. Tackling HWLE inequalities and improving work outcomes for people with osteoarthritis will lead to overall improvements in average HWLE at the national level, which will require an understanding of the links between lower HWLE and key (modifiable and non-modifiable) factors that are potential drivers of health, wellbeing, and work participation.

### Supplementary Information

Supplementary Information 1.

## Data Availability

English Longitudinal Study of Ageing: Data from the English Longitudinal Study of Ageing is freely available to researchers from the UK Data Service. North Staffordshire Osteoarthritis Project: Keele University’s School of Medicine has established data sharing arrangements to support joint publications and other research collaborations upon reasonable request and via our controlled access procedures. Data requests and enquiries should be directed to medicine.datasharing@keele.ac.uk.

## Code availability

Guidance on the use of IMaCh software is available at http://euroreves.ined.fr/imach/wiki/index.php/Documentation.
