# Author Correction: Healthy working life expectancy at age 50 for people with and without osteoarthritis in local and national English populations

**DOI:** 10.1038/s41598-024-62270-1

**Published:** 2024-05-23

**Authors:** Marty Lynch, Milica Bucknall, Carol Jagger, Ross Wilkie

**Affiliations:** 1 School of Medicine, Keele University, Newcastle under Lyme, ST5 5BG UK; 2 MRC Versus Arthritis Centre for Musculoskeletal Health and Work, University of Southampton, Southampton, SO17 1BJ UK; 3 Population Health Sciences Institute, Newcastle University, Newcastle upon Tyne, NE4 5PL UK

Correction to: *Scientific Reports* 10.1038/s41598-022-06490-3, Published online 14 February 2022

The original version of this article contained errors in Table 1, where the values under ‘State 2 (H&NW)’, ‘State 3 (NH&W)’, ‘State 4 (NH&NW) and ‘LE’ in row ‘Has OA’ and ‘No OA’ were incorrect.

The correct and incorrect values appear below.

Incorrect:

**Table:** 

Osteoarthritis	State 2 (H&NW)	State 3 (NH&W)	State 4 (NH&NW)	LE
Has OA	12.17 (11.8, 12.53)	1.62 (1.51, 1.72)	7.53 (7.25, 7.80)	31.31 (30.89, 31.73)
No OA	8.82 (8.36, 9.29)	2.74 (2.48, 3.00)	15.00 (14.43, 15.58)	32.25 (31.59, 32.90)

Correct:

**Table:** 

Osteoarthritis	State 2 (H&NW)	State 3 (NH&W)	State 4 (NH&NW)	LE
Has OA	8.82 (8.36, 9.29)	2.74 (2.48, 3.00)	15.00 (14.43, 15.58)	32.25 (31.59, 32.90)
No OA	12.17 (11.8, 12.53)	1.62 (1.51, 1.72)	7.53 (7.25, 7.80)	31.31 (30.89, 31.73)

Consequently, in the results section,

"For people without osteoarthritis at age 50 in England, HWLE was 10.00 (9.74, 10.26) years, corresponding to 31.0% of LE from age 50 (32.3 [31.6, 32.9] years) (Fig. 2, Table 1). For people with osteoarthritis at age 50 in England, HWLE was 5.68 (5.29, 6.07) years, corresponding to 18.1% of LE from age 50 (31.31 [30.89, 31.73] years) (Fig. 2, Table 1)."

Now reads:

"For people without osteoarthritis at age 50 in England, HWLE was 10.00 (9.74, 10.26) years, corresponding to 31.9% of LE from age 50 (31.31 [30.89, 31.73] years) (Fig. 2, Table 1). For people with osteoarthritis at age 50 in England, HWLE was 5.68 (5.29, 6.07) years, corresponding to 17.6% of LE from age 50 (32.25 [31.59, 32.90] years) (Fig. 2, Table 1)."

The original article has been corrected.

