# Supplementary material for: Healthy working life expectancy at age 50 for people with and without osteoarthritis in local and national English populations
Source: Sci Rep. 2022 Feb 14;12:2408. doi: 10.1038/s41598-022-06490-3 (PMC8844356; doi:10.1038/s41598-022-06490-3)
Supplement: Supplementary file 1 — Supplementary Information 1. [file 41598_2022_6490_MOESM1_ESM.docx]

**Supplementary material: Healthy Working Life Expectancy at age 50 for people with and without osteoarthritis in local and national English populations**

Appendix table 1: Health expectancies for adults in the NorStOP population overall and by osteoarthritis (OA) status

| Population | Sample size | Healthy and in work (1) | Healthy and not in work (2) | Not healthy but in work (3) | Not healthy and not in work (4) | Total life expectancy |
| --- | --- | --- | --- | --- | --- | --- |
| North Staffordshire | 13774 | 6·58 (6·28, 6·87) | 6·96 (6·63, 7·30) | 3·32 (3·09, 3·54) | 12·38 (11·95, 12·80) | 29·23 (28·77, 29·69) |
| OA group | 3260 | 4·31 (3·68, 4·94) | 5·35 (4·72, 5·98) | 3·47 (2·94, 4·01) | 18·69 (17·65, 19·72) | 31·82 (30·98, 32·67) |
| Non-OA group | 10514 | 6·90 (6·57, 7·24) | 7·43 (7·04, 7·83) | 3·22 (2·98, 3·47) | 10·83 (10·38, 11·28) | 28·39 (27·86, 28·92) |

Appendix table 2: Health expectancies from NorStOP sample at age 50 with results of sensitivity analyses

| Population | Interpolation step size | Sample size | Healthy and in work (1) | Healthy and not in work (2) | Not healthy but in work (3) | Not healthy and not in work (4) | Total life expectancy |
| --- | --- | --- | --- | --- | --- | --- | --- |
| Medical record data available (main analysis) | 12 | 13774 | 6·58 (6·28, 6·87) | 6·96 (6·63, 7·30) | 3·32 (3·09, 3·54) | 12·38 (11·95, 12·80) | 29·23 (28·77, 29·69) |
| Medical record data available | **1** | 13774 | 6·90 (6·12, 7·68) | 6·87 (6·53, 7·21) | 3·39 (3·12, 3·65) | 12·11 (11·29, 12·93) | 29·27 (28·78, 29·76) |
| Medical record data available | **Life table** | 13774 |  |  |  |  | 28·99 |
| **Including people with missing medical record data** | 12 | 18413 | 6·52 (6·24, 6·80) | 6·86 (6·54, 7·17) | 3·29 (3·08, 3·50) | 12·2 (11·81, 12·60) | 28·87 (28·44, 29·30) |
| **Including people with missing medical record data** | **1** | 18413 | 6·93 (6·31, 7·55) | 6·76 (6·44, 7·09) | 3·38 (3·14, 3·62) | 11·86 (11·20, 12·52) | 28·93 (28·48, 29·38) |
| **Including people with missing medical record data** | **Life table** | 18413 |  |  |  |  | 28·59 |
